# The systemic inflammation-based Glasgow Prognostic Score as a powerful prognostic factor in patients with upper tract urothelial carcinoma

**DOI:** 10.18632/oncotarget.22641

**Published:** 2017-11-23

**Authors:** Teruo Inamoto, Hideyasu Matsuyama, Shigeru Sakano, Naokazu Ibuki, Kiyoshi Takahara, Kazumasa Komura, Tomoaki Takai, Takuya Tsujino, Yuki Yoshikawa, Koichiro Minami, Kazuhiro Nagao, Ryo Inoue, Haruhito Azuma

**Affiliations:** ^1^ Department of Urology, Osaka Medical College, Osaka, Japan; ^2^ Department of Urology, Graduate School of Medicine, Yamaguchi University, Ube, Japan

**Keywords:** Glasgow Prognostic Score, upper tract urothelial carcinoma, prognosis

## Abstract

**Introduction and Objective:**

The combination of C-reactive protein and albumin, the Glasgow Prognostic Score (GPS), had independent prognostic value in patients with varying cancers, except for upper tract urothelial carcinoma (UTUC). The aim of this study was to describe the relationship between GPS and survival in patients with UTUC after adjustment for other prognostic factors.

**Materials and Methods:**

We queried 2 UTUC databases. Retrospective clinical series on patients with localized UTUC managed by nephroureterectomy with bladder cuff, for whom data from the Yamaguchi Uro-Oncology Group and Osaka Medical College registry, including age, presence of bladder cancer, pT stage, lymphovascular invasion, C-reactive protein (CRP) and albumin, were analyzed. The GPS was constructed by combining CRP and albumin. Cancer specific survival (CSS) and overall survival (OS) and relative excess risk of death were estimated by GPS categories after adjusting for gender, age, ECOG performance status (PS), grade, and lymphovascular invasion (LVI).

**Results:**

Seven hundred and twenty four UTUC patients were identified. Our final cohort included 574 patients; of these, 29.2% died during a maximum follow up of 16.7 years. The estimated mean 10-year CSS of patients with GPS of scre-0, -1, and -2 was 99.5, 95.1, and 75.9 months, respectively. Patients with GPS of score-2 had poorest 10-year estimated mean OS of 67.6 months (57.2–77.9). Raised GPS also had a significant association with excess risk of cancer death at 10 years (GPS 2: Relative Excess Risk = 1.74, 95% CI 1.20–2.54) after adjusting for gender, patients’ age, ECOG PS, and tumor focality. C-index of GPS both for CSS and OS were superior to patients’ age and tumor focality, and comparable to grade.

**Conclusions:**

The GPS is an independent prognostic factor for CSS and OS after surgery with curative intent for localized UTUC. It significantly increases the accuracy of established prognostic factors. The GPS may provide a meaningful adjunct for patient counseling and clinical trial design.

## INTRODUCTION

The knowledge of treatment sequence in daily clinical practice is important for an optimal treatment of upper tract urothelial carcinoma (UTUC). UTUC is uncommon with roughly 3000 cases diagnosed in the United States in 2007, compared to approximately 67000 cases of urothelial carcinoma of the bladder [1]. UTUC accounts for approximately 10% of cancers arising from the kidney and less than 5% of all urothelial malignancies. Several risk factors for developing UTUC have been reported, including delay in radical nephroureterectomy [2] and tumor necrosis [3]. Also, a history of bladder cancer prior to UTUC resection and upper tract tumor multifocality are frequently reported clinical risk factors [4, 5]. Predominantly because of the rarity of the disease, there is limited data to guide clinicians in decision making which consists mostly of small retrospective studies and expert opinion. Candidate pathological predictors, such as E-cadherin, are helpful for improving patient risk stratification [4, 6]. The identification of factors that allow accurate risk stratification of UTUC patients in terms of patients’ survival is emerging, and the area of defining new prognostic markers is of active interest. An unresolved question is the relationship to data from daily clinical practice. This question is becoming valuable in the light of the desire to evaluate the performance of curative surgery for UTUC in daily clinical practice. Prognostic markers in daily practice, including patients’ body composition, age, gender, blood test results, and so on, may offer the opportunity for easily, and reproducible measurement before operation compared to conventional clinicopathological characteristics. The ability to predict which patients will exacerbate while receiving curative surgery would be of great help for clinicians to make adjuvant chemotherapy worthwhile. Previous studies have found that neutrophil-lymphocyte ratio [7, 8], postoperative platelet count [9] from blood test results were predictive of survival in patients with UTUC, but no study has ever assessed the prognostic impact of the Glasgow Prognostic Score (GPS). GPS consists of CRP and albumin as follows; patients with both an elevated CRP level (>1.0 mg/dl) and hypoalbuminemia (< 3.5 g/dl) are allocated a score of 2, patients with only one of these biochemical abnormalities are allocated a score of 1, and patients with neither of these abnormalities are allocated a score of 0. Given the prognostic role of GPS in varying malignancies, including non-small cell lung cancer [10], gastric cancer [11], colorectal liver metastases [12], esophageal cancer [13], plus deep relationship of C-reactive protein in survival of patients with UTUC [14–16, 17], we attempted to verify the prognostic power of the systemic inflammation-based GPS for patients with UTUC with large data across a wide range of disease. In this study we investigated the characteristics of UTUC patients in different GPS groups, and disease risk by GPS, and whether GPS score adjusted for gender, age, ECOG PS, grade, and LVI, has an impact on survival.

## MATERIALS AND METHODS

### Study design and population

Two UTUC databases from the Yamaguchi Uro-Oncology Group [18] and Osaka Medical College registry [19] were combined which include retrospective clinical series on patients with localized UTUC managed by nephroureterectomy with bladder cuff. Two cohorts are approved by the IRB. Of the 724 patients screened for the study, 574 patients were included in the analysis. Synchronous metastatic disease was excluded by chest x-ray, abdominal ultrasonography and computed tomography (CT) of the abdomen and pelvis. No patient had invasive bladder tumor at the time of nephroureterectomy. Previously, open radical nephroureterectomy with open excision of the distal ureter with a bladder cuff was performed to dissect the kidney with the entire length of the ureter and adjacent segment of the bladder cuff. From June 2003 to current, the approach we use is a conventional four-trocar nephrectomy. The hilar and regional lymph nodes adjacent to the ipsilateral great vessel, if possible, were resected. We have measured the CRP and albumin pre-operatively, to be used for the definition of GPS.

### Statistical methods

CSS and OS, as functions of age (discreet and continuous) were evaluated. Patients are followed until death or withdrawal from the study. An event for OS included all deaths within the cohort under investigation, and did not separate those due to the disease of interest from those due to other causes. The Kaplan–Meier method was used to estimate the survival rate and log-rank test was used to compare it among the four age cohorts. Univariate and multivariate analyses were carried out using Cox proportional hazards regression to identify outcome-related factors, after adjustment for categorical (ender, tumor focality, ECOG PS, grade, and pT stage) and continuous variables (age). In precise, we conducted analyses with gender (categorical), age (continuous), tumor focality (unifocal/ multifocal), ECOG PS (0/ 1/ 2/ 3), grade (1/ 2/ 3), and pT stage (pT1/ pT2/ pT3/ pT4). The level of statistical significance was set at *p* < 0.05.

## RESULTS

**Figure 1:**
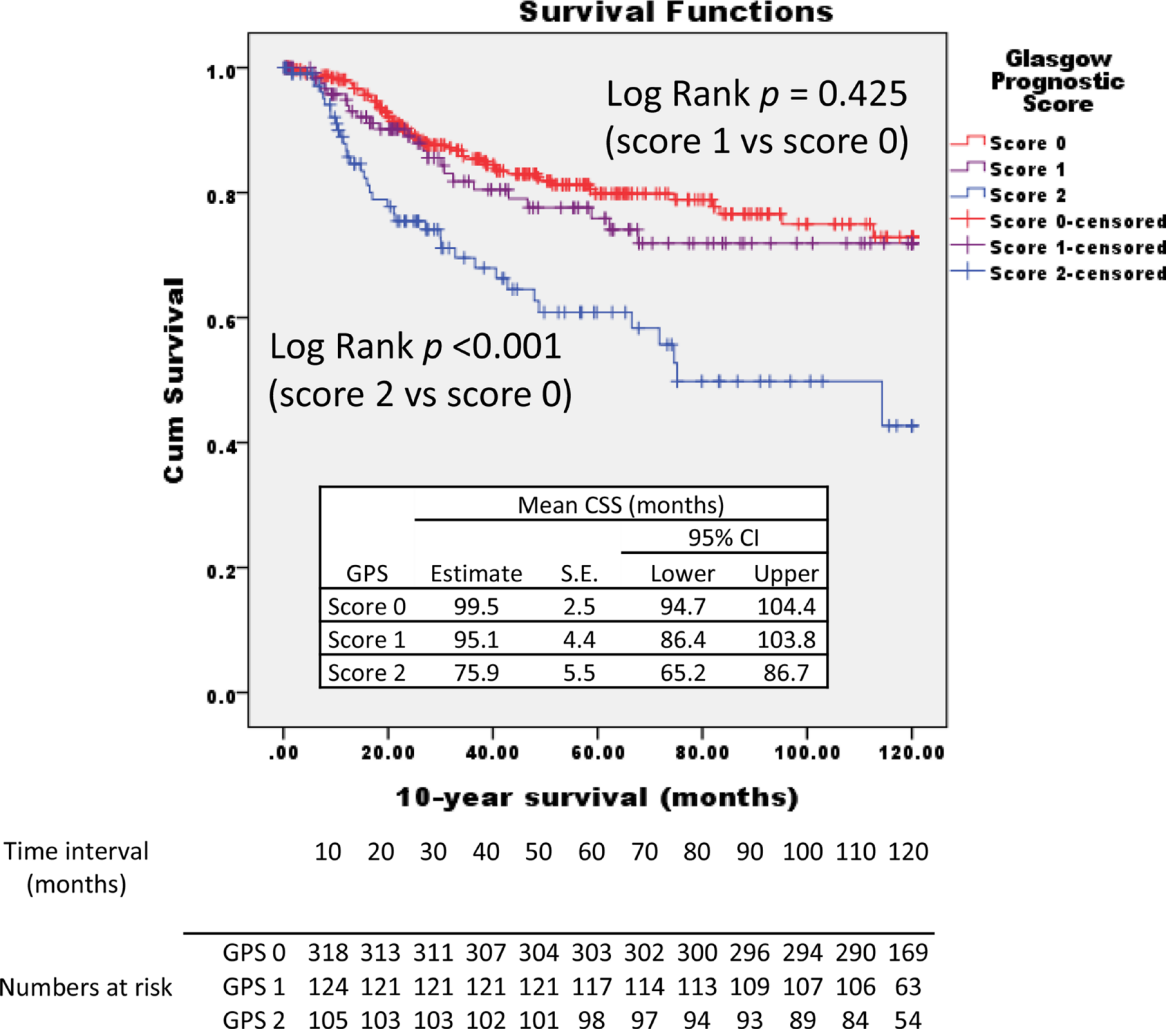
Kaplan–Meier survival curves stratified by Glasgow prognostic score (GPS) The 10-year cancer-specific survival in patients with GPS0, GPS1, and GPS2 are indicated.

**Figure 2:**
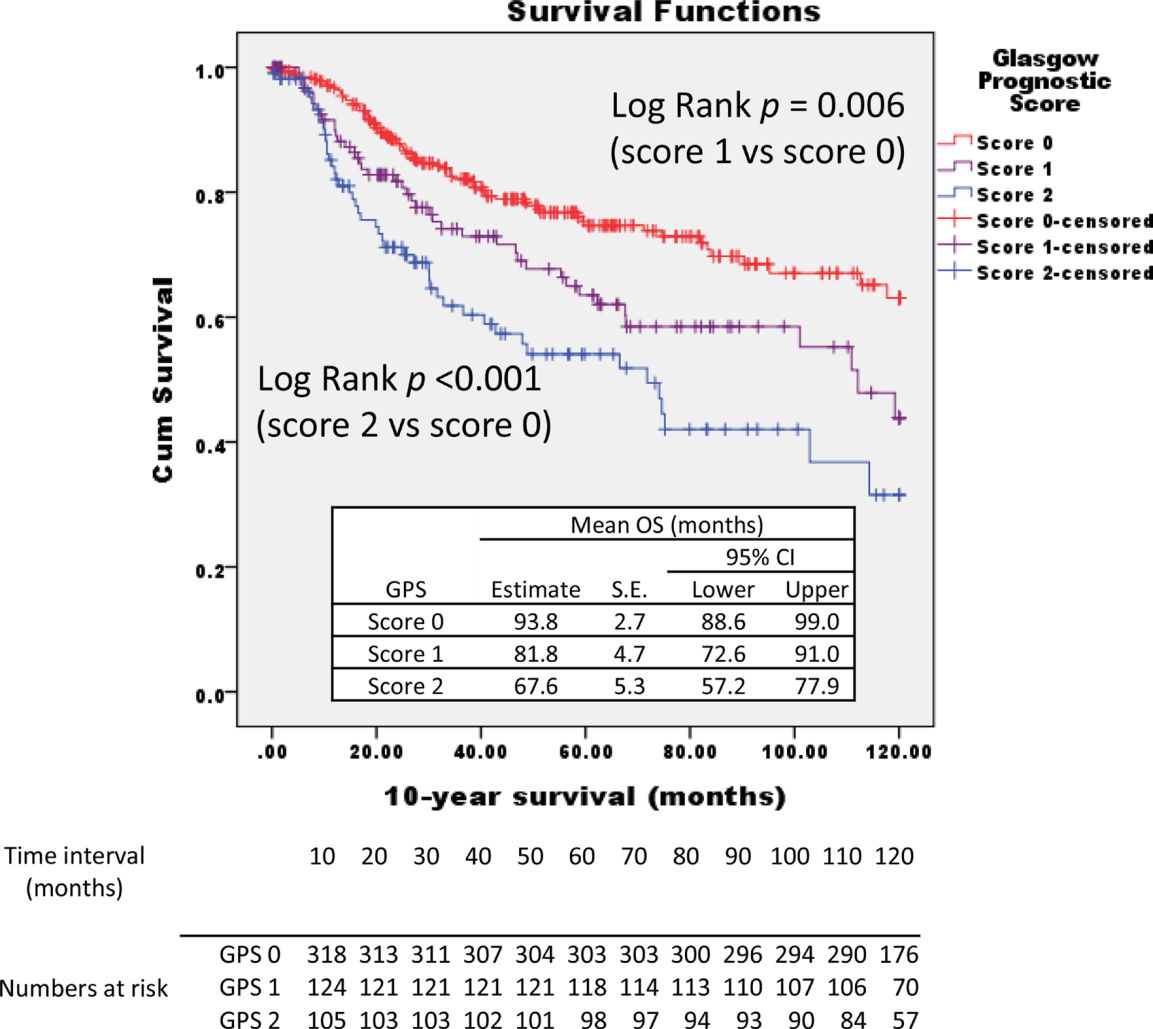
Kaplan–Meier survival curves stratified by glasgow prognostic score (GPS) The 10-year overall survival in patients with GPS0, GPS1, and GPS2 are indicated.

The baseline patient characteristics are shown in Table 1. A total of 574 subjects were identified, 68.8% males with a mean age of 71 years. 96.5% of patients had ECOG PS of 1 or 0. In this cohort, pathological diagnosis was pT1, pT2, pT3 and pT4 in 29.6%, 28.0, 40.1% and 2.3% of patients, respectively, with 42.4% of all patients had advanced disease (≥ T3N0M0). Tumor focality was unifocal in 81.4% of cases (*n* = 467), multifocal in 18.6% (*n* = 107). The relationships between an increasing GPS and patient-related factors are shown in Table 2. There was significant difference in histological pT classification, grade, and LVI between both groups (*p* < 0.05). GPS score 0 patients presented with lower grade, lower pT stage, and larger negative LVI numbers. In 332 patients with score 0; 136 (41.0%) were pT1, 88 (26.5%) were pT2, 104 (31.3%) were pT3, and the remaining 4 (1.2%) were pT4. While in the 110 patients with GPS score 2; 110 (9.1%) were pT1, 2 (1.8%) were pT2, 92 (83.6%) were pT3, and the remaining 6 (5.5%) were pT4. There were thee times as many cases of pT3 and four time the cases of pT4 in GPS score 2 vs score 0. The 5-year and 10-year CSS rates for 574 patients included in the study were 96 ± 1% and 64 ± 3%, respectively. The 5-year and 10-year OS rates were 93 ± 1% and 53 ± 3%, respectively. Kaplan Meier analyses showed that GPS score significantly affected the CSS and OS rates (Figures 1 and 2). The 10-year CSS in patients with GPS0, GPS1, and GPS2 were 69% (reference), 69% (*p* = 0.425), and 47% (*p* < 0.001), respectively (Figure 1). The 10-year OS in patients with GPS0, GPS1, and GPS2 were 63% (reference), 50% (*p* = 0.006), and 45% (*p* < 0.001), respectively (Figure 2).

**Table 1:** Baseline clinico-pathological characteristics

		Number	%
**Gender**	Male	395	68.8%
	Female	179	31.2%
Age (mean, median, min-max)		71, 72, 32–93	
ECOG PS	0	452	78.7%
	1	102	17.8%
	2	15	2.6%
	3	5	0.9%
Tumor focality	Unifocal	467	81.4%
	Multifocal	107	18.6%
pT classification	pT0/pTis/pTa	0	0.0%
	pT1	170	29.6%
	pT2	161	28.0%
	pT3	230	40.1%
	pT4	13	2.3%
Grade	1	38	6.6%
	2	269	46.9%
	3	267	46.5%
LVI	No	385	67.1%
	Yes	189	32.9%

PS, performance status.

**Table 2:** GPS related UTUC: clinico-pathological characteristics

		GPS						
		Score 0		Score 1		Score 2		
		Number	%	Number	%	Number	%	*p* value
Gender	Male	232	69.9%	95	72.0%	68	61.8%	0.19
	Female	100	30.1%	37	28.0%	42	38.2%	
Age (mean, median, min-max)		70, 72, 41–92		72, 72, 46–93		72, 72, 32–93		
ECOG PS	0	269	81.0%	101	76.5%	82	74.5%	0.20
	1	51	15.4%	28	21.2%	23	20.9%	
	2	11	3.3%	1	0.8%	3	2.7%	
	3	1	0.3%	2	1.5%	2	1.8%	
Tumor focality	Unifocal	276	83.1%	104	78.8%	87	79.1%	0.44
	Multifocal	56	16.9%	28	21.2%	23	20.9%	
pT classification	pT1	136	41.0%	24	18.2%	10	9.1%	< 0.01
	pT2	88	26.5%	71	53.8%	2	1.8%	
	pT3	104	31.3%	34	25.8%	92	83.6%	
	pT4	4	1.2%	3	2.3%	6	5.5%	
Grade	1	26	7.8%	8	6.1%	4	3.6%	< 0.01
	2	169	50.9%	66	50.0%	34	30.9%	
	3	137	41.3%	58	43.9%	72	65.5%	
LVI	No	243	73.2%	95	72.0%	47	42.7%	< 0.01
	Yes	89	26.8%	37	28.0%	63	57.3%	

GPS, Glasgow Prognostic Score; UTUC, upper tract urothelial carcinoma; PS, performance status; LVI, lymphovascular invasion

On univariate analysis, age (*p* < 0.01), grade (*p* < 0.01), LVI (*p* < 0.01), pT classification (*p* < 0.01), tumor focality (*p* < 0.01), and GPS (*p* < 0.01) were associated significantly with overall survival (Table 3). On multivariate analysis, increasing grade, LVI, and pT classification were the major predictors of relative excess risk of death at 10 years (Table 3). When the analysis was stratified based on LVI, we observed a significant association between GPS and risk of death within ten years of diagnosis with negative LVI group (GPS score1: OR =1.92, 95% CI 1.18-3.14, *p* = 0.009/ GPS score2: OR = 2.05, 95% CI 1.09-3.84, *p* = 0.03). Also, we observed a significant association between GPS score1 and risk of death within ten years of diagnosis with negative LVI group (OR =1.93, 95% CI 1.17-3.17), but no association with GPS score2 (OR =1.11, 95% CI 0.55-2.25, *p* = 0.78) after adjustment for gender, age, tumor focality, ECOG PS, grade, and pT stage (not shown). As seen in Table 4, GPS remained the solo factor for prognosis when analyzed with non-pathological clinical factors. The C-index for 10-year CSS were 0.51 for age, grade, LVI, pT classification, tumor focality, GPS were, 0.51, 0.66, 0.70, 0.70, 0.54, and 0.58, respectively (Table 5). C-index of GPS both for CSS and OS were superior to patients’ age and tumor focality, and comparable to grade (Table 5). CRP, a constituent element of GPS, is one of the most well-known prognostic factors in urothelial carcinomas including bladder cancer and UTUC [20, 21]. Also, concomitant or metachronous bladder cancer is reported to affect the survival of patients with UTUC [22–24]. We further tested the relationship of GPS with CRP and concomitant bladder cancer for the prognostic impact in patient with UTUC. On multivariate analysis adjusted by CRP, concomitant bladder cancer, and GPS (categorized), GPS remained as a prognostic factor (*p* = 0.034) and GPS score 2 showed highest OR for death (OR = 2.92, 95% CI 0.89-3.01, *p* = 0.01), while CRP (OR =1.04, 95% CI 0.51-2.08, *p* = 0.92) and concomitant bladder cancer (OR = 1.49, 95% CI 0.97-2.28, *p* = 0.07) didn't reach significant odds (data not shown).

**Table 3:** Cox regression analysis for 10-year OS

	Univariate					Multivariate		
	*p* value	OR	95% CI		*p* value	OR	95% CI	
			Lower	Upper			Lower	Upper
Gender	0.56	1.10	0.80	1.52	0.77	0.94	0.64	1.40
Age	< 0.01	1.03	1.02	1.05	0.17	1.02	0.99	1.04
ECOG PS	0.07	1.26	0.98	1.62	0.60	1.09	0.78	1.53
Grade	< 0.01	1.92	1.46	2.53	0.01	1.68	1.14	2.47
LVI	< 0.01	3.54	2.60	4.81	< 0.01	2.90	1.86	4.54
pT classification	< 0.01	1.94	1.60	2.34	< 0.01	1.73	1.29	2.31
Tumor focality	< 0.01	1.70	1.20	2.41	0.12	1.40	0.92	2.14
GPS	< 0.01	1.57	1.31	1.88	0.53	1.07	0.86	1.34

OS, overall survival; CI, confidence interval; PS, performance status; LVI, lymphovascular invasion ; GPS, Glasgow Prognostic Score.

**Table 4:** Cox multivariate regression analysis for 10-year OS

	*p* value	OR	95% CI	
			Lower	Upper
Gender	0.422	1.171	0.797	1.722
Age	0.107	1.017	0.996	1.038
ECOG PS	0.901	1.022	0.721	1.449
Tumor focality	0.058	1.504	0.986	2.294
Glasgow Prognostic Score	< 0.01	1.539	1.241	1.908

OS, overall survival; CI, confidence interval; PS, performance status.

**Table 5:** Model accuracy for 10-year CSS and OS

	CSS					OS				
	C-index	SE	*p*	95% CI		C-index	SE	*p*	95% CI	
				Lower	Upper				Lower	Upper
Age	0.51	0.03	0.72	0.45	0.57	0.55	0.03	0.09	0.50	0.60
Grade	0.66	0.03	< 0.01	0.60	0.71	0.60	0.03	< 0.01	0.55	0.65
LVI	0.70	0.03	< 0.01	0.65	0.76	0.64	0.03	< 0.01	0.59	0.69
pT classification	0.70	0.03	< 0.01	0.65	0.75	0.64	0.03	< 0.01	0.59	0.69
Tumor focality	0.54	0.03	0.14	0.48	0.60	0.55	0.03	0.06	0.50	0.60
GPS	0.58	0.03	< 0.01	0.52	0.65	0.60	0.03	< 0.01	0.55	0.65

CSS, cancer specific survival; OS, overall survival; CI, confidence interval; LVI, lymphovascular invasion ; GPS, Glasgow Prognostic Score

## DISCUSSION

Prognostic inflammatory markers that include cytokines, blood cell composition, and specific proteins have been reported for their value in varying malignancies [25–30]. The relationship between the systemic inflammatory response and nutrition status as an indicator has been previously reported [31–33]. There are papers that investigated the role of GPS in UTUC. Cho et al. investigated the role of GPS in 147 patients with UTUC, with specific interest in bladder recurrence. Multivariate analysis for bladder recurrence after surgery, GPS remained as predictors [34]. Another group investigated the prognostic role of inflammation based biomarkers in 181 patients with UTUC by multivariate analysis and showed that tumor location, pathologic T stage, lymphovascular invasion, margin status, and albumin level were independent contributors for survival [35]. Given these findings, we further verified the prognostic role of GPS in larger cohort with UTUC. Our results have demonstrated that patients with worse GPS score at baseline had significantly shorter survival compared to patients with favorable GPS as illustrated in the Kaplan-Meier survival curves. These results are consistent with studies in other malignancies [36–39]. It is of interest to consider how these results might be combined in a clinical context. At present, patients with high risk features including increased pT stage, high grade, and positive LVI, are offered adjuvant chemotherapy, whereas those patients without high risk features are not. In the present study, among patients with UTUC managed by nephroureterectomy, a high-score GPS indicated a statistically significant poorer prognosis when compared with patients who had a low-score GPS. Such high risk patients may therefore be thought to benefit from adjuvant chemotherapy. The utility of the GPS or modified GPS (mGPS) in predicting the response to chemotherapy is not, to our knowledge, known in UTUC. In contrast, there is evidence that GPS not only identifies those patients who are at an increased risk of recurrent disease in other malignancies [39–46]. Leitch, E.F. et al. showed that TNM stage, monocyte count and mGPS were independently associated with CSS in patients with colorectal cancer [26]. Similarly, Kishiki, T. et al. exhibited that hemoglobin, adjuvant chemotherapy, and mGPS were significant predictive factors for postoperative mortality in patients with incurable stage IV colorectal cancer [43]. Jiang, A.G. et al. found GPS is more accurate than prognostic index (PI) in predicting prognosis for patients with advanced non-small cell lung cancer (NSCLC), with the area under the receiver operating characteristic curve for GPS predicting 1-year disease free survival (DFS) being 0.62, and the area under curve for PI predicting 1-year DFS being 0.57 [47]. Linton, A. et al. investigated the baseline mGPS and NLR in a prospective cohort of chemotherapy-naive patients with metastatic castration-resistant prostate cancer (mCRPC) who received docetaxel and prednisone [42]. The TNM staging system provides the reliable information on patients’ prognosis and aids in the discrimination of patients with early stage disease from those with advanced stage disease. However, it is less accurate for predicting the prognosis of patients with an intermediate extent of tumor invasion. In hour cohort, an increased GPS was associated with increased pT classification (*p* < 0.001), grade (*p* < 0.001), and LVI (*p* < 0.001) but there was no association with gender (*p* = 0.192), ECOG PS (*p* = 0.204), and tumor focality (*p* = 0.441). Moug, S.J. et al. compared two scoring systems which have been proposed in colorectal cancer: the pathologically based positive lymph node ratio (pLNR) and inflammation based mGPS with the TNM staging system in terms of cancer survival [48]. On multivariate model, N category and tumour stage (I-III) were removed from the model, leaving pLNR and mGPS as independent predictors of OS with hazard ratio of 1.51 [48]. Zhang, P et al. investigated the prognostic value of mGPS in patients with inoperable thoracic esophageal squamous cell carcinoma undergoing chemoradiotherapy [49]. They found T stage, M stage and mGPS were independent prognostic indicators for OS, and T stage, M stage, mGPS and platelet count were independent prognostic indicators for PFS, concluding that mGPS can be used in combination with conventional TNM staging to predict survival in patients with squamous cell carcinoma undergoing chemoradiotherapy [49]. The present study has limitations that are typical to the study based on retrospective database, which include the risk of unidentified confounding factors, missing data, and the potential for miscoding of data. Additionally, the present cohort does not describe some important pathologic factors such as updated grading system, perineurial tumor invasion, the status of previous or concomitant urinary bladder lesions, the histological patterns, mitotic activity, and so on. Even with these limitations, our data present the new characterization of UTUC and outcomes.

Therefore, on the basis of the available evidence, the GPS/mGPS may potentially be included, together with the other known factors, in the post-operative follow-up of patients with primary operable UTUC and the stratification of patients entering treatment of adjuvant chemotherapy.

## CONCLUSIONS

The present study is the first to demonstrate that malnutrition, as determined by GPS was significant independent predictors of survival in patients with UTUC and had a strong association with LVI. Nutritional and inflammatory statuses are modifiable factors which may be favorably altered by early nutritional and anti- inflammatory interventions, thereby resulting in a significant improvement in survival. These promising and rather simple interventions need to be investigated further to fully understand their impact on the clinical outcomes of patients with UTUC.
